# Development of a theory-informed questionnaire to assess the acceptability of healthcare interventions

**DOI:** 10.1186/s12913-022-07577-3

**Published:** 2022-03-01

**Authors:** Mandeep Sekhon, Martin Cartwright, Jill J. Francis

**Affiliations:** 1 School of Health Sciences, City University of London, Northampton Square, London, EC1V 0JB UK; 2 Department of Population Health Sciences, Faculty of life Sciences and Medicine, Kings College London, London, SE1 1UL UK; 3 Melbourne School of Health Sciences, University of Melbourne, Melbourne, VIC 3010 Australia; 4 Centre of Implementation Research, Ottawa Hospital Research Institute – General Campus, 501 Smyth Road, Ottawa, ON K1H 8L6 Canada

**Keywords:** Acceptability, Questionnaire development, Pre-validation methods, Healthcare intervention, Theoretical framework

## Abstract

**Background:**

The theoretical framework of acceptability (TFA) was developed in response to recommendations that acceptability should be assessed in the design, evaluation and implementation phases of healthcare interventions. The TFA consists of seven component constructs (affective attitude, burden, ethicality, intervention coherence, opportunity costs, perceived effectiveness, and self-efficacy) that can help to identify characteristics of interventions that may be improved. The aim of this study was to develop a generic TFA questionnaire that can be adapted to assess acceptability of any healthcare intervention.

**Methods:**

Two intervention-specific acceptability questionnaires based on the TFA were developed using a 5-step pre-validation method for developing patient-reported outcome instruments: 1) item generation; 2) item de-duplication; 3) item reduction and creation; 4) assessment of discriminant content validity against a pre-specified framework (TFA); 5) feedback from key stakeholders.

Next, a generic TFA-based questionnaire was developed and applied to assess prospective and retrospective acceptability of the COVID-19 vaccine. A think-aloud method was employed with two samples: 10 participants who self-reported intention to have the COVID-19 vaccine, and 10 participants who self-reported receiving a first dose of the vaccine.

**Results:**

1) The item pool contained 138 items, identified from primary papers included in an overview of reviews. 2) There were no duplicate items. 3) 107 items were discarded; 35 new items were created to maximise coverage of the seven TFA constructs. 4) 33 items met criteria for discriminant content validity and were reduced to two intervention-specific acceptability questionnaires, each with eight items. 5) Feedback from key stakeholders resulted in refinement of item wording, which was then adapted to develop a generic TFA-based questionnaire.

For prospective and retrospective versions of the questionnaire, no participants identified problems with understanding and answering items reflecting four TFA constructs: affective attitude, burden, perceived effectiveness, opportunity costs. Some participants encountered problems with items reflecting three constructs: ethicality, intervention coherence, self-efficacy.

**Conclusions:**

A generic questionnaire for assessing intervention acceptability from the perspectives of intervention recipients was developed using methods for creating participant-reported outcome measures, informed by theory, previous research, and stakeholder input. The questionnaire provides researchers with an adaptable tool to measure acceptability across a range of healthcare interventions.

**Supplementary Information:**

The online version contains supplementary material available at 10.1186/s12913-022-07577-3.

## Statement of contribution

What is known on this topic:

- It is important to assess the acceptability of healthcare interventions.
- A recently developed theoretical framework of acceptability (TFA) proposes seven component constructs (affective attitude, burden, ethicality, intervention coherence, opportunity costs, perceived effectiveness, and self-efficacy) to help identify characteristics of interventions that may be improved.

What this study adds:

- A generic, theoretically informed questionnaire for assessing acceptability of healthcare interventions has been developed.
- The comprehensibility, relevance, and answerability of the adapted items in the generic acceptability questionnaire were assessed using think-aloud methods.
- The generic TFA-based questionnaire is a brief and adaptable tool for researchers and clinicians to measure intervention/treatment acceptability across a range of healthcare settings.

## Introduction

A health intervention that is not acceptable to the people who deliver or receive it is difficult to evaluate, as intervention facilitators are unlikely to deliver the key components faithfully and recipients are unlikely to engage with the intervention as required. Intervention acceptability from the perspective of patients and healthcare professionals has been proposed to have an impact on intervention implementation, uptake, adherence, intended outcomes and overall effectiveness [1–5].

The UK Medical Research Council (MRC) has published guidance for intervention developers and researchers, describing best practice methods for designing and evaluating complex interventions [4–6]. The guidance recommends that researchers assess intervention acceptability [5, 6] but provides few suggestions about how to do this.

In a systematic overview of reviews, we found that 55% of included reviews used behavioural measures as indicators of acceptability [7], such as total trial dropout rate and reasons for discontinuation [8–10]. However, there are several reasons, other than acceptability problems, that may explain why participants discontinue treatment or withdraw from an intervention [11]. Reasons may include lack of motivation to take part, mistrust of the research process [12] or personal circumstances external to the trial or the intervention [13]. Furthermore, reliance on measures of observed behaviour does not provide information on which aspects of an intervention are (un)acceptable and hence does not inform how to enhance acceptability. Notably, in our overview of reviews, we found that there was no standardised or validated intervention acceptability questionnaire [7].

The Theoretical Framework of Acceptability (TFA) [7, 14] can guide assessments of intervention acceptability across three temporal perspectives (before, during and after participation in an intervention) and from the perspectives of intervention deliverers and recipients. In some contexts, a questionnaire approach may offer a time-efficient way to identify potential problems with intervention acceptability, within all four phases of intervention development and evaluation identified in the MRC guidance, i.e., development, feasibility and piloting, evaluation, implementation [4–6].

Questionnaires are often considered a practical and cost-effective method for assessing participant outcomes (e.g., quality of life, emotional health, experienced symptoms). They have the advantage of being able to be administered to large samples, and to enable quantitative analysis including longitudinal assessments and direct comparisons between different trial arms and across different studies [15, 16].

To assess whether other researchers had developed a TFA-based acceptability questionnaire, we completed a forward citation search in Google scholar (October 2021), and identified two articles that report using quantitative measures of the TFA constructs [17, 18]. Neither article reported using an established method to develop their questionnaire, and neither questionnaire included an item assessing overall acceptability. Keyworth et al. [18] describe useful methods for analysing data from Likert scale responses to TFA items but reported problems with the wording of the Opportunity Costs item and potential issues with the response format of this item and the Self-Efficacy item. Renko et al’s [17] questionnaire contained several examples where the item wording did not accurately reflect the TFA definitions or items that conflated multiple TFA constructs. This study included no assessment of the comprehensibility of the items to participants. These studies highlight the challenges of developing a TFA questionnaire and demonstrate the need for a generic TFA questionnaire that can be used as the starting point for questionnaire development across health interventions.

The development and validation of questionnaires is a complex process [19]. Common methods to develop questionnaires include both inductive “bottom-up” approaches and deductive “top-down” approaches. The bottom-up approach focuses on generating items from empirical data often generated by exploratory qualitative methods (e.g., semi-structured interviews, focus groups) to ensure items represent the perspectives of the target population [20]. The top-down approach focuses on reviewing the literature to identify existing items [20, 21] or generating items based on pre-existing theory.

To develop the questionnaire, we combined methods for the development and pre-validation of participant-reported outcome measures [20] and methods used to establish the content validity of items in theoretically informed questionnaires [22] (see Method section and supplementary files 1 and 2 for details).

### Aims and objectives

The aim of the current study was to develop a generic TFA questionnaire that can be adapted to assess acceptability of any healthcare intervention. The objectives were to:

1. adopt the 5-step pre-validation Patient reported Outcomes (PRO) method [20] and the Discriminant Content Validation (DCV) method [22] to develop two preliminary acceptability questionnaires based on the TFA (one for healthcare professionals and one for patients);
2. optimise the two preliminary questionnaires using feedback from key stakeholders on comprehensibility and relevance of items;
3. assess the comprehensibility, relevance and answerability of items in the generic acceptability questionnaire using think-aloud methods.

## Methods

### Context of preliminary questionnaires

#### Healthcare professional acceptability questionnaire

The first questionnaire was developed to assess acceptability to intervention recipients (healthcare professionals) of two feedback interventions delivered as part of the AFFINITIE Research Programme which aimed to optimise audit-and-feedback processes to improve blood transfusion practice [23, 24]. Intervention 1 consisted of feedback reports that were “enhanced”, compared with usual feedback delivered to hospital staff. Intervention 2 consisted of “follow-on support” (a web-based toolkit and telephone support) provided to hospital transfusion teams to help them plan their response to the feedback reports [25].

#### Patient acceptability questionnaire

The second questionnaire was developed to assess acceptability to intervention recipients of a new appointment-booking system. Recipients were patients who regularly attended an eye clinic for management of Benign Essential Blepharospasm (BEB) and Hemifacial Spasm (HFS) [26]. There were two conditions: standard care (i.e. appointments booked for patients by the clinic at approximately regular intervals) and the new service model (patient-initiated appointment booking service where patients called a nurse helpline for an appointment when their symptoms necessitated it). Trial participants were randomly allocated to either the standard service or patient-initiated appointment services for 9 months.

### Step 1: item generation

Supplementary file 2 presents further details of the 5–step pre validation PRO method. To generate a pool of potentially relevant items, we adapted an empirical approach to selecting systematic review papers and their included primary papers, based on the findings from our overview of systematic reviews [7] to determine how others have approached the issue of assessing acceptability. In our overview of reviews, the findings indicated that review authors assessed acceptability either via measures of observed behaviour, self-reported measures, or a combination of both. Next, an inclusive approach was applied to the review papers, and their included primary papers that reported using self-report measures to assess acceptability (or related constructs) if authors stated acceptability had been assessed via:

- A measure of satisfaction
- Reasons for discontinuation
- Qualitative open-ended interviews
- User perspectives and evaluations of the intervention

Reviews that identified assessing acceptability via ‘observed measures of behaviour’ were not reviewed or included in our item pool as they would not have been relevant to our primary aim of developing a generic TFA questionnaire.

Primary papers that reported assessing acceptability, as described above, were eligible if they met any of following inclusion criteria:

- Exact item wording and response format is presented in the text or in the appendix or supplementary file of the paper
- Wording of interview questions used to assess acceptability are reported in the paper
- Descriptions of reported reasons for discontinuation reflected assessments of acceptability reported in the paper, e.g., reasons for discontinuation provided by participants included side effects of treatment [27]; preference for treatment choice [28]
- Descriptions of user perspectives and evaluations applied to assess intervention acceptability are reported in the text of the paper (e.g., evaluations of programme acceptability among programme planners, policy makers and members of the community) [29].

Information relating to the origin of the item, response format, content and wording of interview questions and descriptions of reasons for dropout, user perspectives and evaluations was entered into an Excel database (the item pool).

### Step 2: Deduplication

Extracted items were reviewed for the three types of duplication by one author as suggested by Prior et al. (2011) [20] (Supplementary file 2).

### Step 3: item reduction and item creation

Two researchers independently reviewed items extracted in Step 1 and removed items:

- If items were specific to an intervention and non-generalisable (e.g., do you follow a special diabetes diet?);
- If reasons for discontinuation and descriptions of user perspectives and evaluations of an intervention could not be reworded as a question (e.g., “loss to follow up, other reasons”).

To maximise coverage of the TFA constructs, one author drafted new items based on the definitions of the seven TFA constructs (Table 1) for the healthcare professional questionnaire and the patient questionnaire. The new items were specific to each intervention, and the temporal perspective was also represented in item wording. For example, in the BEB/HFS questionnaire, not all TFA constructs were appropriate for assessing the acceptability of the standard service (control condition). Participants receiving standard care did not perform a behaviour (i.e., book their own appointment) because the next appointment was scheduled by their treating healthcare professional [26]. Thus, the constructs of burden and self-efficacy were not relevant. The response options of the new items also reflected the TFA constructs (Table 1).

**Table 1:**
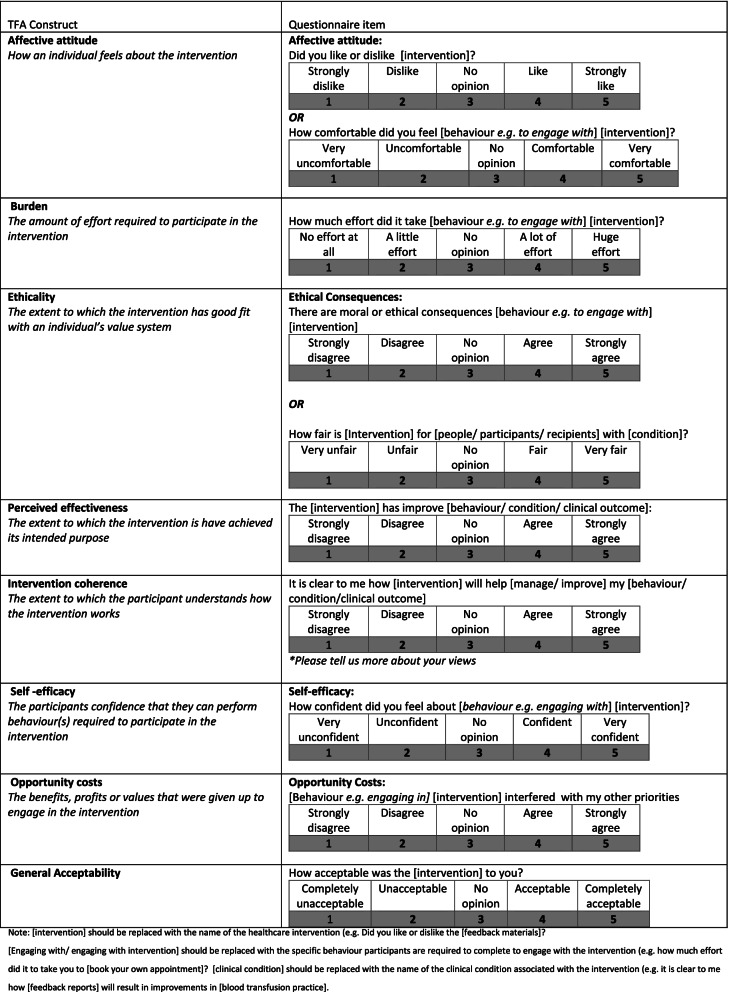
Generic form of TFA acceptability questionnaire

### Step 4: assessment of content coverage against a pre-existing theoretical framework

To test the discriminant content validity of items against the seven TFA constructs, the DCV method was applied [22, 30]. Previous research on the number of judges required for judgement tasks suggests between 2 and 20 [31, 32]. All members of the Psychology Group within the Centre of Health Services Research at City University London were invited to take part. Eight members agreed to participate as expert judges, including four PhD students, three postdoctoral research fellows, one research assistant and one senior lecturer. Participants were provided with the construct definitions and an Excel table of 65 items to be classified. Five judges received a paper copy of the Excel table to complete in a face-to-face session; three elected to receive electronic versions of these materials and instructions on how to complete the DCV task.

### Step 5: feedback on preliminary version of acceptability questionnaire from key stakeholders

Prior et al. (2011) [20] recommend conducting a think-aloud study with members of the target population to provide feedback on a newly developed questionnaire. Due to time constraints of both trials, this was not possible. Instead, two principal investigators (with clinical backgrounds) working on the AFFINITIE programme, and two patient representatives on the BEB/HFS study steering group, were asked to provide feedback on the draft questionnaire, which was emailed to them together with an invitation to read and comment on each item for comprehensibility and relevance.

### Development of a generic TFA questionnaire

By reviewing both TFA questionnaires, a generic (not intervention-specific) acceptability questionnaire was developed. Item wording that was common to both the intervention-specific questionnaires was reviewed to assess whether the specific intervention descriptions and behavioural descriptions could be replaced with equivalent generic terms and phrases, “[intervention]” and “[engage with the intervention]”, so that all items could be applicable to a range of healthcare interventions (examples presented in Results section below).

The generic version of the questionnaire was adapted to assess perceptions of both prospective and retrospective acceptability of the COVID-19 vaccine. The questionnaire consisted of 9 items: one item reflecting each of the TFA constructs of affective attitude, burden, perceived effectiveness, intervention coherence, self-efficacy, and opportunity costs, two items reflecting ethicality, and one general acceptability item. Both ethicality items were included in the questionnaire, as we were not certain which of the two items would be most comprehensible and answerable.

Whilst the TFA identifies seven component constructs of acceptability, we propose a generic TFA questionnaire should include an item to assess overall acceptability, for two main reasons. First, by including a general acceptability item, researchers are able to explore which of the seven TFA constructs influences or drives participants’ general acceptability judgment and secondly, it allows researchers to determine evaluations of overall acceptability of an intervention, which cannot be inferred from the sub-constructs alone (owing to uncertainties about the relative weightings of the items).

#### Think-aloud study

A ‘think-aloud’ study was conducted between March–April 2021 to explore people’s views on the comprehensibility, relevance, and answerability of the items in two versions of questionnaires to assess the acceptability of a COVID-19 vaccine. Full ethical approval was obtained from King’s College London Research Ethics Committee (REF:MRA-20/21–22,254).

Participants consisted of a convenience sample of 20 individuals recruited via Twitter. Ten individuals self-reported having received a first dose of the COVID-19 Vaccine (for the retrospective version of the acceptability questionnaire) and 10 self-reported an intention to have the COVID-19 Vaccine in the near future (for the prospective version of the acceptability questionnaire). Participants expressed their interest to take part in the study by contacting the primary researcher via e-mail. An information sheet and a consent form were then e-mailed to each participant. Participants signed the consent form and e-mailed back a scanned copy to the primary researcher.

Each participant completed the TFA questionnaire via a synchronous video call (Microsoft Teams), supported by a researcher (MS), who provided verbal instructions adapted from the think-aloud studies reported by French et al. (2007) [33] and Green and Gillhooly (1996) [34]. Participants were instructed to read each question, verbalise their thoughts (i.e., think-aloud) whilst completing the questionnaire, and to provide their response for each item. Participants were also instructed to state when a question item did not make sense, or if they were not sure what the item was asking. After the think-aloud interview, the researcher asked participants to provide more details about any items identified as problematic and asked participants their opinions about the questionnaire in general (e.g., length; ease or difficulty of completing the questionnaire). The whole procedure took a maximum of half an hour per participant. Interviews were audio-recorded via the Microsoft Teams software, and transcribed verbatim.

Data were analysed by assigning each response to an adapted version of the categories applied by French et al. 2007 [33]:

1. No signification problems identified
2. Participant reread question, or seriously stumbled (i.e., stammered or stuttered because of misreading) in answering it (problems in understanding question),
3. Difficulty generating an answer
4. Questioned content of item (identified problems with how the question was worded, = did not understand the question), or
5. Answered a different question from the one that was asked or gave reasoning inconsistent with the answer given (problems in comprehending/answering question, misinterpretation of question).

To assess the reliability of the researcher’s (MS) coding, two additional researchers (JF and MC) each completed double coding on two transcripts (i.e., four transcripts in all). Agreement in coding between MS and MC, and between MS and JF was registered if the same part of a transcript was independently coded into the same category. Disagreement was registered where one researcher coded a section of text, but the other researcher did not, or else coded it into a different category. Percentage agreement rather than Cohen’s Kappa was used to assess reliability because the items (i.e., sentences in transcripts) may have been coded into more than one of the categories [35, 36]. Any disagreements in coding were discussed and changes were agreed that would be applied to subsequent coding of the remaining transcripts.

## Results

**Fig. 1:**
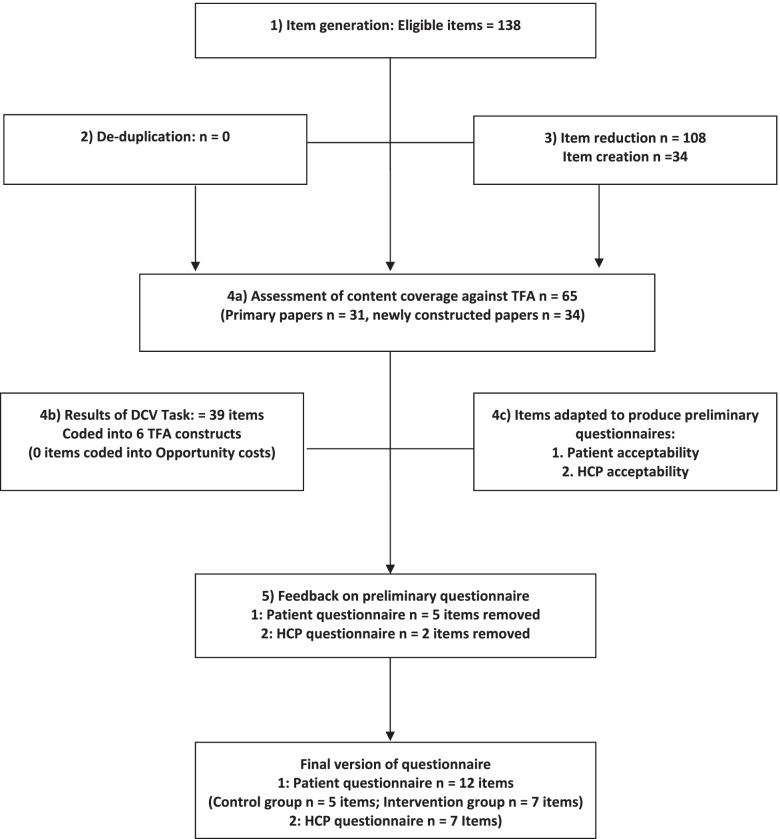
Adapted 5 step PRO methodology Flowchart applied to test content validity of the theoretical framework and to develop the patient and HCP acceptability questionnaires

Figure 1 presents an overview of the adapted 5-Step PRO methodology applied to develop the two TFA questionnaires. Results of each of step are described in detail below.

### Step 1: item generation

Twelve systematic reviews identified primary papers that had applied self-report assessment measures to investigate acceptability [7]. These included: three reviews assessing acceptability via measures of satisfaction [37, 38, 39]; four reviews describing reasons for discontinuation as indicators of acceptability [27, 40, 41, 42]; two reviews using participants’ perspectives and evaluations as assessments of intervention acceptability [29, 43]; two reviews synthesising evidence from open questions to assess acceptability [44, 45], and one review assessing acceptability via participants’ attitudes [46].

Three hundred and forty-three primary papers included potential measures of acceptability. Of these, 325 (95%) papers were retrieved (18 were unavailable). Of the 325, 290 (89%) articles did not meet the inclusion criteria for extraction of items. One hundred and thirty-eight items were extracted from 35 papers.

### Step 2: De-duplication

There were no literal duplications of items, or differences in content (e.g. timeframe) or overlap with other generic items that were to be included in the questionnaires. Thus, no items were removed at this stage.

### Step 3: item reduction and refinement of item wording

Two authors read each of the 138 items, applied the inclusion criteria (presented in Methods section above), and agreed on decisions to remove 107 items. Based on the construct definitions, one author drafted 34 new items, 17 items relating to the AFFINITIE trial [23, 24] and 17 items relating to the standard care and patient-initiated appointment booking services within the BEB/HFS trial [26].

### Step 4: assessment of content coverage against a pre-existing theoretical framework

Eight participants completed the DCV task on 65 items (31 identified from the primary reviews and 34 new items). Within DCV tasks, content validity is usually tested using single sample t-tests (based on judges’ rating of their confidence that the item represents a specified construct) [22, 30]. In the current study this would have required 455 (i.e., 65 (no. of items) x seven (number of construct definitions)) one-sample t-tests based on data from eight judges. The likely number of Type I errors was a substantive threat to validity, therefore null hypothesis significance testing was deemed inappropriate.

Instead, the analysis focused on descriptive statistics (means, standard deviations and medians, interquartile ranges) for each item. A median confidence rating across judges of + 5 or greater (on the scale of − 10 to + 10) was considered an appropriate threshold to avoid lengthy questionnaires and taken as an indication that the judges agreed that an item closely reflected a construct.

Thirty-nine of the 65 items had a median confidence rating of 5 or greater and were coded into six of the TFA constructs, with no identified items for the construct, opportunity costs. Six items had a median confidence rating of 5 or greater for more than one construct, thus these items did not achieve discriminant validity [22].

The remaining 33 items (Supplementary file 3) were reviewed by all three authors for adaptation and inclusion in a preliminary version of the AFFINITIE acceptability questionnaire and BEB/HFS questionnaire (Supplementary files 4 and 5). A key decision included selecting only one item per construct, and the item that could be best adapted for both preliminary questionnaires taking into account the response scale. The research team applied the following criteria when selecting items for both preliminary versions of the questionnaire:

(i) Degree to which each item reflected the core definition of the construct;
(ii) Degree to which the wording of each item was clear and unambiguous;
(iii) Degree to which the wording of the item was appropriate for the intervention.

Disagreements were resolved by discussion. Thus, for each intervention-specific questionnaire eight items were included, one item per TFA construct and one general acceptability item. The rationale for including only a single item per construct was to keep the TFA questionnaires as brief as possible to enhance the questionnaires’ usability, ease and speed of completion, and to make it more feasible to include an assessment of acceptability alongside other outcome measures.

### Step 5: feedback on preliminary version of acceptability questionnaire from key stakeholders

#### AFFINITIE trial

The two principal investigators of the AFFINITIE programme advised that the TFA items would be more user-friendly and reduce participant burden for completion if the response anchors on the TFA items could be adapted to reflect the same 5-point Likert scales for the other items in the broader process evaluation questionnaire (i.e., strongly agree - strongly disagree).

Considering this feedback, the TFA response scale were changed to 5-point Likert scales, rather than the original scales. Supplementary file 4 displays the original TFA questionnaire (version 1) and the modified TFA questionnaire (version 2) applied in the AFFINITIE trial.

#### BEB and HFS trial

Feedback from the two patient representatives on the draft version of the BEB/HFS questionnaire resulted in the re-wording of three items to improve clarity. Both patient representatives suggested incorporating an option for additional comments for the intervention coherence item. Supplementary file 5 displays the final version of the control group and intervention group TFA-informed acceptability questionnaires for the BEB/HFS trial.

### Generic TFA questionnaire

Table 1 displays a generic form of each of the items that can be adapted to assess intervention acceptability. There are two example items for the constructs of ethicality to reflect the differences in patient and healthcare professional interventions. Each item can be adapted to reflect a specific healthcare intervention by inserting a description of ‘the intervention’ (e.g. “feedback materials”) or to include the specific behaviour required to engage with the intervention (e.g. “booking own treatment appointment”). For some constructs, the item may include a reference to both the specific behaviour required to engage with the intervention and description of the intervention. For example, for the AFFINTIE trial, the generic burden item ‘How much effort did it take to [engage with intervention]?’ was adapted to ‘How much effort did it take to read the feedback materials?’, where ‘read’ is the behaviour and ‘feedback materials’ is the description of the intervention.

#### Think-aloud study

Table 2 presents an overview of the problems identified for each of the prospective and retrospective TFA items adapted to assess acceptability of the COVID-19 vaccine. Stemler (2004) [36] suggests that when using percentage agreements to assess inter-rater reliability, values from 75 to 90% indicate an acceptable level of agreement. The agreement between the primary researcher (MS) and each of the additional researchers (MC, JF) was good (80–100%).

**Table 2:**
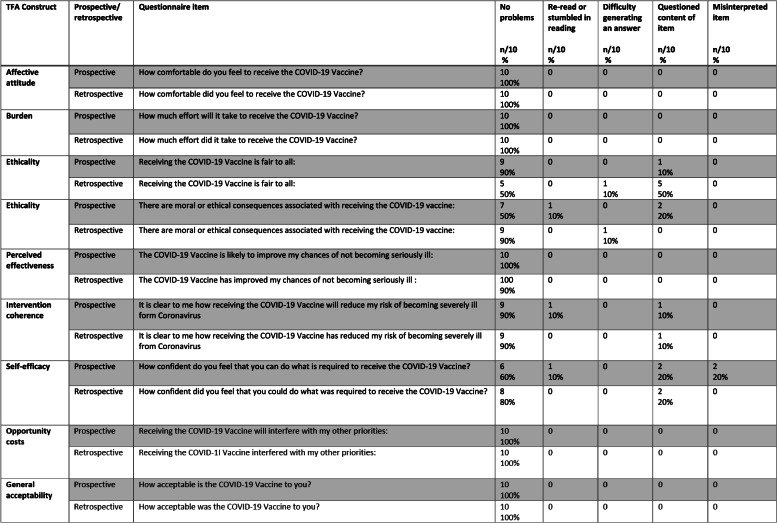
Frequency and type of problems identified for each of the prospective and retrospective TFA items adapted to assess acceptability of the COVID-19 vaccine

Supplementary file 6 provides illustrative examples of the problems that participants reported for the ethicality, intervention coherence and self-efficacy items. No participant identified problems with understanding and answering five out of nine items reflecting overall acceptability and four constructs: affective attitude, burden, perceived effectiveness, opportunity costs. Most problems that participants encountered included questioning the content of both prospective ethicality items (fairness item (*n* = 1); moral consequences item (*n* = 2)), retrospective ethicality (moral consequences item (*n* = 5)), both intervention coherence items (prospective (*n* = 1), retrospective (*n* = 2)) and self-efficacy items (prospective item (*n* = 2); retrospective item *n* = 2)). Participants re-read three items for the prospective questionnaire. This included the items for the constructs of ethicality (fairness) (*n* = 1), intervention coherence (*n* = 1) and self-efficacy (*n* = 1). Some participants also had difficulties in generating an answer for the retrospective ethicality items (fairness *n* = 1), moral consequences *n* = 1). Misinterpretations occurred for the prospective self-efficacy item (*n* = 2).

## Discussion

This study has described the development of a generic questionnaire, based on the TFA, for assessing the acceptability of healthcare interventions. We adapted the generic TFA-based questionnaire to assess prospective and retrospective acceptability of the COVID-19 vaccine in the UK context.

Our think-aloud findings indicate that the generic items assessing affective attitude, burden, perceived effectiveness and opportunity costs, adapted to assess acceptability of the COVID-19 vaccine, were comprehensible and answerable to all participants in our sample. Our think-aloud study, however, did identify some issues with the items representing the constructs ethicality and self-efficacy. This suggests that each of the ethicality items may not make sense in relation to some interventions. On reflection, the self-efficacy items may have been clearer if we had specified the behaviour more clearly (e.g. booking your COVID-19 vaccine appointment or attending your booked appointment). We advise researchers to pilot test their entire questionnaire with members of their intended population in relation to any specific intervention and adapt it, if participants find the items difficult to answer.

### Strengths and limitations

The 5-step pre-validation method [20] provided a systematic approach in which both inductive (existing items from the overview of reviews) [7] and deductive methods (definitions of each of the seven TFA component constructs) were applied to develop two intervention-specific acceptability questionnaires. For each version of the preliminary questionnaire, the TFA was the basis for development of new items to reflect the construct, opportunity costs, as none of the items assessing this construct in the DCV exercise achieved discriminant content validity. Explicit TFA construct definitions were also important in re-wording existing items for inclusion in the acceptability questionnaires. Another strength of this study is the application of the DCV method to assess the content validity and discriminant validity of the identified existing and newly generated items across all seven component constructs in the TFA. As recommended, the DCV method [22] was completed in the early phase of developing the TFA-informed acceptability questionnaires. A further strength was the seeking of stakeholder feedback for each of the two preliminary TFA questionnaires (one involving healthcare professionals and one involving patients), which resulted in minor amendments to the questionnaires.

The present study also demonstrates strengths in its comprehensive think-aloud study to assess the adaptation of both the prospective and retrospective acceptability items to assess acceptability of the COVID-19 vaccine. The study included an adequate sample size of 20 participants (10 participants per questionnaire), which allowed us to assess the face validity and answerability of the items and identify specific problems for each of the items.

There were several limitations. In this study, the large pool of items and the limited pool of judges meant it was not possible to complete the recommended statistical analysis for the DCV method [30] as multiple hypothesis testing would have generated too many false positives. However, the use of descriptive statistics and a threshold confidence rating to determine eligibility for inclusion in the questionnaire, identified an adequate number of items with good evidence of discriminant content validity. The pre-validation methodology recommends completing a think-aloud study with the target population on a newly developed questionnaire [20]. Whilst efforts were made to gain feedback from two stakeholders per preliminary questionnaire, it was not possible to complete think-aloud interviews with participants from both trial contexts that may have provided further information with regards to the comprehensibility, relevance and answerability of the draft questionnaires. Lastly, whilst efforts were made to recruit an adequate number of participants for the generic acceptability questionnaire think-aloud interviews, participants were recruited from a convenience sample, thus limiting the generalisability of the findings.

### Recommendations for future work

Whilst systematic methods have been applied to develop the two TFA-based questionnaires, and the generic items have been applied to assess the acceptability of the COVID 19 vaccine, with a think aloud study completed, further work will be needed to establish further psychometric properties of both questionnaires. This is true of all pre-validation phases in developing new measures [20, 22]. We provide a supplementary file (Supplementary file 7) with guidance on how to adapt each of the items, and some notes on suggestions for analysing the TFA generic questionnaire.

## Conclusion

This study has described the systematic methods applied to develop two intervention-specific acceptability questionnaires based on the recently developed Theoretical Framework of Acceptability (TFA), which have been adapted to develop a generic TFA-based questionnaire. We offer the generic TFA-based questionnaire as a brief and adaptable tool for researchers and clinicians to measure intervention acceptability across a range of healthcare interventions and to contribute to establishing an evidence base for psychometric properties of the items.

## Supplementary Information

**Additional file 1.**

**Additional file 2.**

**Additional file 3.**

**Additional file 4.**

**Additional file 5.**

**Additional file 6.**

**Additional file 7.**

## Data Availability

The datasets used and/or analysed during the current study available from the corresponding author on reasonable request.

## Abbreviations

TFA: Theoretical Framework of Acceptability
PRO: Patient Reported Outcomes
DCV: Discriminant Content Validation
BEB: Benign Essential Blepharospasm
HSF: Hemifacial Spasm
AFFINITIE: Audit and Feedback INterventions to Increase evidencebased Transfusion practIcE
COVID-19: Coronavirus disease
