# Supplementary material for: Development of a theory-informed questionnaire to assess the acceptability of healthcare interventions
Source: BMC Health Serv Res. 2022 Mar 1;22:279. doi: 10.1186/s12913-022-07577-3 (PMC8887649; doi:10.1186/s12913-022-07577-3)
Supplement: Supplementary file 1 — Additional file 1. [file 12913_2022_7577_MOESM1_ESM.docx]

**Supplementary file 1, description of the PRO development methodology and the Discriminant Content Validation (DCV) methodology.**

**Patient Reported Outcomes (PRO) development methodology**

To enhance the reporting of top-down methods for item generation and reduction, Prior et al (2011) proposed a 5–step method for the pre-validation stages of PRO patient reported outcome measures instrument development. Step 1 involves item generation via literature review; the relevant literature is reviewed to identify existing items which are evaluated against eligibility criteria. Step 2 involves identifying duplicate items identified in Step 1, which are discarded if they are literal duplicates, reflect the same conceptual content themes, or overlap with items in other instruments/questionnaires that will be administered alongside the new questionnaire. Step 3 involves item reduction; some remaining items identified in Step 2 are removed at the macro level (items associated with content themes not relevant to the new measure) and then at the micro level (applying criteria specific to study context to select items for inclusion). In Step 4, all remaining items remaining after Step 3 are assessed for conceptual content coverage against a “pre-existing theoretical framework appropriate to the objectives of the instrument and the target population” (p. 2). Step 4 thus provides an assessment of the content validity of the items in the new instrument. Lastly, Step 5 involves exploratory pilot testing with the target population via think-aloud methods to gain insights on the new instrument, specifically on “comprehensibility, acceptability, relevance and answerability” (p.4).

Key reference:

- Prior ME, Hamzah JC, Francis JJ, Ramsay CR, Castillo MM, Campbell SE, et al. Pre-validation methods for developing a patient reported outcome instrument. *BMC medical research* *methodology*. 2011; **11**: 112

**Description of the Discriminant Content Validation (DCV) Method**

The DCV method involves six stages: (1) identify or generate definitions for each of the constructs that the items aim to measure; (2) identifying items from existing measures and/or generating new items to develop an item pool for the new measure; (3) identify judges (experts in the theoretical constructs being assessed or members of population for which the measure has been designed); (4) judges use a rating scale (e.g. -10 to +10) to indicate how confident they are that an item reflects the target construct; (5) statistical tests such as single-sample t-tests (or Wilcoxon signed rank test) are applied to identify whether judges’ confidence ratings are significantly different from zero(to test content validity); (6) the discriminant validity of each item is evaluated to identify items that assess only one construct and discriminate between potentially competing constructs. This is done by checking “whether an item has a stronger rating for the relevant target construct than for competing constructs using paired t-tests or Wilcoxon signed-rank tests” (p.243, Johnston et al., 2014).

Key papers describing the DCV method:

- Johnston M, Dixon D, Hart J, Glidewell L, Schröder C, Pollard B. Discriminant content validity: A quantitative methodology for assessing content of theory-based measures, with illustrative applications. *British journal of health psychology*. 2014; **19**: 240-57.
- Dixon D, Johnston M, McQueen M. The Disabilities of the Arm, Shoulder and Hand Questionnaire (DASH) can measure the impairment, activity limitations and participation restriction constructs from the International Classification of Functioning, Disability and Health (ICF). *BMC* *musculoskeletal disorders*. 2008; **9**: 114.
