# Supplementary material for: Development of a theory-informed questionnaire to assess the acceptability of healthcare interventions
Source: BMC Health Serv Res. 2022 Mar 1;22:279. doi: 10.1186/s12913-022-07577-3 (PMC8887649; doi:10.1186/s12913-022-07577-3)
Supplement: Supplementary file 2 — Additional file 2. [file 12913_2022_7577_MOESM2_ESM.docx]

**Supplementary file 2: 5-Step Pre-validation Methodology developed by Prior et al (2011) and adapted steps applied to develop two TFA-based questionnaires**

| Step | 5 Step PRO development methodology | Adapted step applied to generate pool of acceptability items |
| --- | --- | --- |
| 1 | **Item generation**   1. Systematic identification of existing PRO instruments meeting eligibility criteria 2. Selection of additional instruments (e.g. generic instruments) to be administrated alongside the new PRO instrument 3. All items from the identified instruments form the initial item pool (to which steps 2-5 are applied) | **Item generation**   1. Identifying primary papers from systematic review papers that stated an assessment measure of acceptability was applied 2. Extracting items from primary papers based on eligibility criteria 3. All items from identified quantitative and qualitative measures from the initial item pool |
| 2 | **Item deduplication**  Items are deleted if   1. They are literal duplications (identically worded items, or duplication of item content) 2. Their content differs only by timeframe or attribution to a condition of interest (e.g. do you have difficulty because of your condition) 3. Their content overlaps with generic measures to be administrated alongside new instruments (e.g. SF-36) | **Item Deduplication**  Step not applicable as no duplicated items identified |
| 3 | **Item Reduction**   1. Macro level: Item discarded with content themes (dimensions of health) that are not appropriate for inclusion in the new instrument (e.g. treatment satisfaction) 2. Micro level: application of explicit study-relevant criteria to select items for conclusion in draft instrument (actual content area) | **Item Reduction and Item creation**   1. Macro level: removal of items that are specific to an intervention or condition and not generalisable (e.g. Score the dressing comfort and its aesthetic acceptance) and if the item cannot be reworded 2. Generation of new items to assess the acceptability of two complex interventions. The items focused on the definitions of each of the constructs within the Theoretical Framework of Acceptability (TFA) |
| 4 | **Assessment of content coverage against a pre –existing theoretical framework**  (revisit 3E if content coverage suboptimal) | **Assessment of content coverage against a pre –existing theoretical framework**  Assessed each of the items (extracted and newly generated) against the TFA by applying principles of the DCV method (Dixon et al. 2008; Johnston et al. 2014) |
| 5 | **Exploratory pilot work with target population**  To assess comprehensibility, acceptability, relevance and answerability in order to inform instrument refinement (item removal &/or re-wording) (e.g. ‘think aloud’ study, focus groups | **Feedback on preliminary version of acceptability questionnaire from stakeholders/ patients**  Health care professional questionnaire: Clinician feedback  Patient acceptability questionnaire: patient representative feedback |
