# Supplementary material for: Development of a theory-informed questionnaire to assess the acceptability of healthcare interventions
Source: BMC Health Serv Res. 2022 Mar 1;22:279. doi: 10.1186/s12913-022-07577-3 (PMC8887649; doi:10.1186/s12913-022-07577-3)
Supplement: Supplementary file 3 — Additional file 3. [file 12913_2022_7577_MOESM3_ESM.docx]

**Supplementary file 3: 33 Items that achieved a median confidence rating of 5 or greater to indicate discriminant content validity in this study**

| Construct items | Response format | Median  Standard Deviation |
| --- | --- | --- |
| **Affective attitude (8 items)** | | |
| What do you think was the best and worst part of [intervention]? | Open text written response | 18  8.79 |
| I was happy with the [ intervention] | 5-point scale:  Agree very strongly- disagree very strongly | 16  6.67 |
| What did you particularly like about [ intervention]? | Open text written response | 16  6.62 |
| I liked using [intervention] | 5-point scale:  Agree very strongly- disagree very strongly | 16  6.67 |
| Did you like [engaging with the intervention]? | 5-point scale:  Strongly disagree- Strongly agree | 18  1.88 |
| Did you like or dislike the [name of the intervention]?? | 5-point scale:  Strongly disagree- Strongly agree | 18  7.08 |
| How much will you look forward to [engaging with the intervention]? | 5-point scale:  Not at all - A lot | 16  8.33 |
| How much would you like [having your treating healthcare professional continuing to behaviour e.g. book your appointment]? | 5-point scale:  Not at all - A lot | 16  8.43 |
| **Burden (5 items)** | | |
| During the past 4 weeks, how often were you bothered by the side effects from [intervention]? | a) all of the time, b) most of the time c) some of the time d) a little of the time e) none of the time | 15  8.39 |
| During the past 4 weeks, how often did you have problems [getting your behaviour e.g. prescription filled]? | a) all of the time, b) most of the time c) some of the time d) a little of the time e) none of the time | 15  8.39 |
| How much time did it take to [behaviour e.g. read name of the intervention]? | 5-point scale:  Not a lot of time - A lot of time# | 15  6.7 |
| How much time did it take you to [work through and apply the name of the intervention]? | 5-point scale:  Not a lot of time - A lot of time | 20  7.12 |
| How easy do you think it will be to attend [intervention]? | 5-point scale:  Very easy – very difficult | 15  8.56 |
| **Ethicality (3 items)** | | |
| For me to [engage with the intervention]is the right thing to do | 5-point scale:  Strongly disagree- Strongly agree | 15  8.22 |
| Using the [intervention] has ethical implications for patient care | 5-point scale:  Strongly disagree- Strongly agree | 20  3.66 |
| How fair do you feel [intervention] will be? | 5-point scale:  Not at all fair- very fair | 15  6.35 |
| **Perceived effectiveness (7 items)** | | |
| how much do you think you[ improved with respect to][intervention]? | 0 (not at all) to 7 (very much so) | 19  2.18 |
| how much has your life changed, in terms of [engaging with intervention] | 0 (not at all) to 7 (very much so) | 15  2.03 |
| If future [intervention] are presented in a similar format, how effective do you think they will be [in making a difference to clinical practice]? | 5-point scale:  Not at all effective- very effective | 20  7.02 |
| How effective do you think [intervention] would be in making [a difference to clinical practice]? | 5-point scale:  Not at all effective- very effective | 20  6.99 |
| How effective do you think [e engaging with the intervention] would be? | 5-point scale:  Not at all effective- very effective | 17  6.61 |
| How effective do you think it will be to have your [appointments scheduled by your healthcare team]? | 5-point scale:  Not at all effective- very effective | 18.5  6.77 |
| How effective do you think it would be for your [treating healthcare professional to continue to book your appointment]? | 5-point scale:  Not at all effective- very effective | 18.5  6.67 |
| **Intervention coherence (4 items)** | | |
| It makes sense to me how [intervention] will result in [improvements in patient care] | 5-point scale:  Strongly disagree- Strongly agree | 15  3.3 |
| It makes sense to me how using [intervention] result in [improvements in patient care] | 5-point scale:  Strongly disagree- Strongly agree | 15  8.1 |
| It is clear to me how [intervention] would help me manage my [clinical condition/symptoms] | 5-point scale:  Strongly disagree- Strongly agree | 18  4.25 |
| it is clear to me how having [intervention – control arm] would help me manage my [clinical condition/symptoms condition] | 5-point scale:  Strongly disagree- Strongly agree | 18.5  6.86 |
| **Self-efficacy (6 items)** | | |
| During the past 4 weeks, how often were you unable to do [what was necessary to follow your doctors’ treatment plans for your [clinical condition/symptoms]? | a) all of the time, b) most of the time c) some of the time d) a little of the time e) none of the time | 16.5  8.55 |
| In the last week how, many days out of 7 were you able to follow [intervention]? | 0=7 days | 15.5  2.56 |
| In the last week how, many days out of 7 were you able to follow your [intervention]? | 0-7 days | 15  7.66 |
| I feel confident that I can [engage with intervention] | 5-point scale:  Very unconfident- very confident | 15.5  7.73 |
| how confident do you feel about [engaging with the intervention]? | 5-point scale:  Very unconfident- very confident | 15  8.2 |
| How easy do you think it will be to [engage with the intervention]? | 5-point scale:  Very difficult- very easy | 16.5  8.56 |
| **Opportunity costs (0 items)** | | |
