# Supplementary material for: Development of a theory-informed questionnaire to assess the acceptability of healthcare interventions
Source: BMC Health Serv Res. 2022 Mar 1;22:279. doi: 10.1186/s12913-022-07577-3 (PMC8887649; doi:10.1186/s12913-022-07577-3)
Supplement: Supplementary file 4 — Additional file 4. [file 12913_2022_7577_MOESM4_ESM.docx]

**Supplementary file 4: Version 1 of the TFA informed acceptability questionnaire and version 2 of the TFA informed acceptability questionnaire applied in the process evaluation phase of the AFFINITIE research programme**.

|  | V1 | V2 applied in AFFINITIE trial |
| --- | --- | --- |
| Global acceptability | To what extend did you find the PBM elective audit materials acceptable?   \| Completely unacceptable \| Unacceptable \| No opinion \| Acceptable \| Completely acceptable \| \| --- \| --- \| --- \| --- \| --- \| \| 1 \| **2** \| **3** \| **4** \| **5** \| | The feedback materials were acceptable:   \| Strongly disagree \| Disagree \| No opinion \| Agree \| Strongly agree \| \| --- \| --- \| --- \| --- \| --- \| \| 1 \| **2** \| **3** \| **4** \| **5** \| |
| Affective attitude  *How an individual feel about the intervention* | Did you like reading the feedback materials?   \| Strongly dislike \| Dislike \| No opinion \| Like \| Strongly like \| \| --- \| --- \| --- \| --- \| --- \| \| 1 \| **2** \| **3** \| **4** \| **5** \| | The feedback materials were interesting to read:   \| Strongly disagree \| Disagree \| No opinion \| Agree \| Strongly agree \| \| --- \| --- \| --- \| --- \| --- \| \| 1 \| **2** \| **3** \| **4** \| **5** \| |
| Burden  *The amount of effort that was required to participate in the intervention* | How much effort did it take you to read the materials?   \| No effort at all \| A little effort \| Same effort \| A lot of effort \| Huge effort \| \| --- \| --- \| --- \| --- \| --- \| \| 1 \| **2** \| **3** \| **4** \| **5** \| | It required effort for me to read the feedback materials:   \| Strongly disagree \| Disagree \| No opinion \| Agree \| Strongly agree \| \| --- \| --- \| --- \| --- \| --- \| \| 1 \| **2** \| **3** \| **4** \| **5** \| |
| Ethicality  *The extent to which the intervention has good fit with an individual’s value system* | Are there any other moral or ethical consequences of the materials?   \| Substantial negative  consequences \| Negative consequences \| No consequences \| Positive consequences \| Substantial positive consequences \| \| --- \| --- \| --- \| --- \| --- \| \| 1 \| **2** \| **3** \| **4** \| **5** \|   Please tell us more about your views | There are moral or ethical consequences to the feedback materials:   \| Strongly disagree \| Disagree \| No Opinion \| Agree \| Strongly agree \| \| --- \| --- \| --- \| --- \| --- \| \| 1 \| **2** \| **3** \| **4** \| **5** \| |
| Perceived Effectiveness  *The extent to which the intervention is perceived to have achieved its intended purpose* | How effective were the feedback materials in supporting improvements in patient care?   \| Not at all effective \| Ineffective \| No Opinion \| Effective \| Very effective \| \| --- \| --- \| --- \| --- \| --- \| \| 1 \| **2** \| **3** \| **4** \| **5** \| | The feedback materials are likely to improve patient care:   \| Strongly disagree \| Disagree \| No Opinion \| Agree \| Strongly agree \| \| --- \| --- \| --- \| --- \| --- \| \| 1 \| **2** \| **3** \| **4** \| **5** \| |
| Opportunity costs  *the benefits, profits or values that were given up to engage in the intervention* | Did reading the feedback materials interfere with your other priorities?   \| Strongly disagree \| Disagree \| No Opinion \| Agree \| Strongly agree \| \| --- \| --- \| --- \| --- \| --- \| \| 1 \| **2** \| **3** \| **4** \| **5** \| | Reading the feedback materials interfered with my other priorities:   \| Strongly disagree \| Disagree \| No Opinion \| Agree \| Strongly agree \| \| --- \| --- \| --- \| --- \| --- \| \| 1 \| **2** \| **3** \| **4** \| **5** \| |
| Self-efficacy  *The participant's confidence that they can perform the behaviour(s) required to participate in the intervention* | How confident are you that you can identify the most relevant information for your hospital from the feedback materials?   \| Very unconfident \| Unconfident \| No opinion \| Confident \| Very confident \| \| --- \| --- \| --- \| --- \| --- \| \| 1 \| **2** \| **3** \| **4** \| **5** \| | I am confident that I can identify the most relevant information for my site from the feedback materials:   \| Very unconfident \| Unconfident \| No opinion \| Confident \| Very confident \| \| --- \| --- \| --- \| --- \| --- \| \| 1 \| **2** \| **3** \| **4** \| **5** \| |
| Intervention coherence  *The extent to which the participant understands the intervention and how it works* | It makes sense to me how the feedback materials will result in improvements in patient care:   \| Strongly disagree \| Disagree \| No opinion \| Agree \| Strongly agree \| \| --- \| --- \| --- \| --- \| --- \| \| 1 \| **2** \| **3** \| **4** \| **5** \|   Please tell us more about your views | It makes sense to me how the feedback materials will result in improvements in patient care:   \| Strongly disagree \| Disagree \| No opinion \| Agree \| Strongly agree \| \| --- \| --- \| --- \| --- \| --- \| \| 1 \| **2** \| **3** \| **4** \| **5** \|   Please tell us more about your views |
|  | If you have any additional thoughts regarding the feedback materials, please can you write them here: | If you have any additional thoughts regarding the feedback materials, please can you write them here: |
