# Supplementary material for: Development of a theory-informed questionnaire to assess the acceptability of healthcare interventions
Source: BMC Health Serv Res. 2022 Mar 1;22:279. doi: 10.1186/s12913-022-07577-3 (PMC8887649; doi:10.1186/s12913-022-07577-3)
Supplement: Supplementary file 5 — Additional file 5. [file 12913_2022_7577_MOESM5_ESM.docx]

**Supplementary file 5: The final version of both the control group and intervention group TFA informed acceptability questionnaires applied in the BEB and HFS trial**

|  | Intervention group | Control group |
| --- | --- | --- |
| Global acceptability | How acceptable would it be to book your own appointments?   \| Completely unacceptable \| Unacceptable \| No opinion \| Acceptable \| Completely acceptable \| \| --- \| --- \| --- \| --- \| --- \| \| 1 \| **2** \| **3** \| **4** \| **5** \| | How acceptable would it be for your treating Healthcare Professional to continue booking your appointments?   \| Completely unacceptable \| Unacceptable \| No opinion \| Acceptable \| Completely acceptable \| \| --- \| --- \| --- \| --- \| --- \| \| 1 \| **2** \| **3** \| **4** \| **5** \| |
| Affective attitude  *How an individual feel about the intervention* | How much would you like booking your own appointments?   \| Strongly dislike \| Dislike \| No opinion \| Like \| Strongly like \| \| --- \| --- \| --- \| --- \| --- \| \| 1 \| **2** \| **3** \| **4** \| **5** \| | How much would you like having your appointments booked for you by your Treating Healthcare Professional?   \| Strongly dislike \| Dislike \| No opinion \| Like \| Strongly like \| \| --- \| --- \| --- \| --- \| --- \| \| 1 \| **2** \| **3** \| **4** \| **5** \| |
| Burden  *The amount of effort that was required to participate in the intervention* | How much effort do you think it would be to book your own appointments?   \| No effort at all \| A little effort \| No opinion \| A lot of effort \| Huge effort \| \| --- \| --- \| --- \| --- \| --- \| \| 1 \| **2** \| **3** \| **4** \| **5** \| | n/a |
| Perceived Effectiveness  *The extent to which the intervention is perceived to have achieved its intended purpose* | How likely is that you would attend appointments that you booked yourself?   \| Very unlikely \| Unlikely \| No opinion \| Likely \| Very likely \| \| --- \| --- \| --- \| --- \| --- \| \| 1 \| **2** \| **3** \| **4** \| **5** \| | How likely is it that you would attend the appointments booked for you by your Treating Healthcare Professional?   \| Very unlikely \| Unlikely \| No opinion \| Likely \| Very likely \| \| --- \| --- \| --- \| --- \| --- \| \| 1 \| **2** \| **3** \| **4** \| **5** \| |
| Ethicality  *The extent to which the intervention has good fit with an individual’s value system* | How fair (to all patients) is a system where patients book their own appointments?   \| Very unfair \| Unfair \| No opinion \| Fair \| Very fair \| \| --- \| --- \| --- \| --- \| --- \| \| 1 \| **2** \| **3** \| **4** \| **5** \| | How fair (to all patients) is the current system where appointments are booked by the Treating Healthcare Professional?   \| Very unfair \| Unfair \| No opinion \| Fair \| Very fair \| \| --- \| --- \| --- \| --- \| --- \| \| 1 \| **2** \| **3** \| **4** \| **5** \| |
| Opportunity costs  *the benefits, profits or values that were given up to engage in the intervention* | Booking my own appointments would interfere with my other priorities:   \| Strongly disagree \| Disagree \| No opinion \| Agree \| Strongly agree \| \| --- \| --- \| --- \| --- \| --- \| \| 1 \| **2** \| **3** \| **4** \| **5** \| | \| Strongly disagree \| Disagree \| No opinion \| Agree \| Strongly agree \| \| --- \| --- \| --- \| --- \| --- \| \| 1 \| **2** \| **3** \| **4** \| **5** \|   Having my appointments booked for me by my treating Healthcare Professional would interfere with my other priorities: |
| Self-efficacy  *The participant's confidence that they can perform the behaviour(s) required to participate in the intervention* | How confident would you feel about booking your own appointments?   \| Very unconfident \| Unconfident \| No opinion \| Confident \| Very confident \| \| --- \| --- \| --- \| --- \| --- \| \| 1 \| **2** \| **3** \| **4** \| **5** \| | n/a |
| Intervention coherence  *The extent to which the participant understands the intervention and how it works* | It is clear to me how booking my own appointments would help me manage my eye condition   \| Strongly disagree \| Disagree \| No opinion \| Agree \| Strongly agree \| \| --- \| --- \| --- \| --- \| --- \| \| 1 \| **2** \| **3** \| **4** \| **5** \|   Please tell us more about your views | It is clear to me how having my appointment booked for me by my Treating Healthcare Professional would help me manage my eye condition.   \| Strongly disagree \| Disagree \| No opinion \| Agree \| Strongly agree \| \| --- \| --- \| --- \| --- \| --- \| \| 1 \| **2** \| **3** \| **4** \| **5** \|   Please tell us more about your views |
