# Supplementary material for: Development of a theory-informed questionnaire to assess the acceptability of healthcare interventions
Source: BMC Health Serv Res. 2022 Mar 1;22:279. doi: 10.1186/s12913-022-07577-3 (PMC8887649; doi:10.1186/s12913-022-07577-3)
Supplement: Supplementary file 6 — Additional file 6. [file 12913_2022_7577_MOESM6_ESM.docx]

**Supplementary file 6**

The following examples illustrate the problems that occurred for the four of the items, reflecting 3 TFA constructs.

##### **Ethicality items**

The prospective ethicality item assessing ‘fairness’ generated one problem in the think aloud interviews. One participant had difficulty in understanding the context of the item. For example:

*“ Receiving the COVID-19 Vaccine is fair to all. What do you mean by that? I don’t understand what you mean by fairness. Do you mean its fair everyone is offered the vaccine or fair that everyone has a choice? I’m not sure – so don’t know how to answer this question” (Participant 005, Prospective questionnaire)*

Two participants also reported difficulties in understanding the context of the prospective ethicality item, assessing ‘moral consequences’. For example:

“*There are moral or ethical consequences associated with receiving the COVID-19 Vaccine. What do you mean by that? I’m not sure if I understand this question if I’m honest. If it’s to do with actually rolling out vaccine, I don’t think there are any issues. But I’m not sure if that’s what you mean. So, I’m not too sure.” (Participant 006, Prospective Questionnaire)*

Five participants that completed the retrospective version of the TFA questionnaire, also reported problems in understanding the fairness ethicality item. For example:

*“Fair to all. fair in what respect? Fair as in priority related? Is it fair that higher age groups and the most vulnerable are being prioritised first? Or do you mean is it fair that people are being invited to have the vaccine at the right time? Or is it being accessible to all ethnic minorities? Or is it fair that some people have access to mobile phones and the internet so they can book? It’s bit ambiguous, I don’t know what you mean by fair, so I can’t really answer this question.” ( Participant 013, Retrospective questionnaire).*

##### **Intervention coherence**

The prospective intervention coherence item generated two problems for one participant. The participant re-read the item a number of times, and also had difficulties in understanding the context of the item:

*“ Number 6. It is clear to me how receiving the COVID-19 Vaccine will reduce my risk of becoming severely ill from coronavirus. It is clear to me. Is it clear to me that receiving the vaccine will reduce my risk.. urm..is it clear to me? I don’t know..is it clear to me? I don’t know. I know … I guess the figures are saying…actually I don’t really know what you’re asking here.” (Participant 010, Prospective questionnaire)*

One participant also had difficulties in understanding the context of the retrospective intervention coherence item.

“ so, number 6. It is clear to me how *receiving the COVID-19 Vaccine has reduced my risk of becoming severely ill from coronavirus. Hmm. I think that’s quite a similar question to number 5. I’m not sure that I can see the difference. Well, the difference I can see in it is that it’s clear to me. So I guess is it saying to me how clear it is to understand how the vaccine has reduced my risk. Which I don’t understand because I haven’t a clue on how the vaccine actually works. As I’ve not really paid attention on how the vaccine works. So, I’m not sure on that question.” ( Participant 019, Retrospective questionnaire).*

##### **Self-efficacy**

Two participants did not understand the prospective self-efficacy item. Both participants had to re-read the question a number of times and informed the researcher that they did not understand the question thus were unable to answer it. For example:

*“How confident do you feel you can do what is required to receive the COVID- 19 Vaccine? Hmm, let me read this again. How confident do you feel you can do what is required to receive the COVID- 19 Vaccine I don’t really understand the question, so not sure how to answer this question.” (Participant 007, Prospective questionnaire).*

Two additional participants also misinterpreted the prospective self-efficacy item. For instance:

“ *How confident do you feel you can do what is required to receive the COVID- 19 Vaccine? I have limited control over what I can do. I guess it all depends on your age, and if you have any underlying health conditions. But I suppose there are certain things you can do. You could go to a walk-in centre, to see if they have any spare vaccines. Or if you’re a carer you can be escalated to get it sooner. So yeah there is some control you have. What is the question asking? So yeah, for that I’m saying do I have control in getting the vaccine. so yeah I think that really depends on the government’s plans and what age bracket you fall under” (Participant 008, Prospective questionnaire).*

With regards to the retrospective self-efficacy item, two out of the 10 participants also reported difficulties in understanding the context of the item. For example:

*“I’m not sure what that means. Does it mean the actual logistics to getting to the vaccination centre or my own ability to have the vaccine? or are you asking if I have confidence in the vaccine working for me personally? ’m not sure what you mean by this question.” ( Participant 016, Retrospective questionnaire).*
