# Supplementary material for: Development of a theory-informed questionnaire to assess the acceptability of healthcare interventions
Source: BMC Health Serv Res. 2022 Mar 1;22:279. doi: 10.1186/s12913-022-07577-3 (PMC8887649; doi:10.1186/s12913-022-07577-3)
Supplement: Supplementary file 7 — Additional file 7. [file 12913_2022_7577_MOESM7_ESM.docx]

**Supplementary file 7**

**TFA Generic Questionnaire – Guidance and notes from authors**

The items in table 1 are based on our operationalisation of each of the TFA constructs. When adapting the items, it is important to take into careful consideration if each of the items are relevant to the specific intervention and context. Whilst we recommend all seven TFA items and the General acceptability item should be considered, in some contexts all items will not be applicable.

We recommend researchers use their judgment and consult with their stakeholder advisors. We also recommend that once the items have been adapted, the questionnaire is piloted as per standard methods.

For all items in Table 1:

- [intervention] should be replaced with name of the healthcare intervention/service
- [behaviour] should be replaced with specific behaviour participants are required to complete to engage with the intervention.
- [behaviour/ clinical condition/ clinical outcome] should be replaced with the primary outcome associated with intervention
- [people/ participants/ recipients] – the appropriate term should be selected depending on context of intervention

| TFA construct | Generic TFA questionnaire items | Notes |
| --- | --- | --- |
| **Affective attitude**  *How an individual feels about the intervention* | Did you like or dislike [intervention]?   \| Strongly dislike \| Dislike \| No opinion \| Like \| Strongly like \| \| --- \| --- \| --- \| --- \| --- \| \| 1 \| **2** \| **3** \| **4** \| **5** \|   ***OR***  How comfortable did you feel [behaviour *i.e. to engage with*] [intervention]?   \| Very uncomfortable \| Uncomfortable \| No opinion \| Comfortable \| Very comfortable \| \| --- \| --- \| --- \| --- \| --- \| \| 1 \| **2** \| **3** \| **4** \| **5** \| | Depending on the context of the intervention and behaviours associated with the intervention *one* of the two items should be used.  In some contexts, ‘like or dislike’ will not be appropriate, thus ‘comfortable’ may be more appropriate. |
| **Burden**  *The amount of effort required to participate in the intervention* | How much effort did it take [behaviour *i.e. to engage with*] [intervention]?   \| No effort at all \| A little effort \| No opinion \| A lot of effort \| Huge effort \| \| --- \| --- \| --- \| --- \| --- \| \| 1 \| **2** \| **3** \| **4** \| **5** \| |  |
| **Ethicality**  *The extent to which the intervention has good fit with an individual’s value system* | How fair is [Intervention] for [people/ participants/ recipients] with [condition]?   \| Very unfair \| Unfair \| No opinion \| Fair \| Very fair \| \| --- \| --- \| --- \| --- \| --- \| \| 1 \| **2** \| **3** \| **4** \| **5** \|   ***OR***  There are moral or ethical consequences [behaviour *i.e. to engage with*] [intervention]   \| Strongly disagree \| Disagree \| No opinion \| Agree \| Strongly agree \| \| --- \| --- \| --- \| --- \| --- \| \| 1 \| **2** \| **3** \| **4** \| **5** \| | Depending on the context of the intervention and behaviours associated with the intervention *one* of the two items should be used. |
| **Perceived effectiveness**  *The extent to which the intervention is perceived to have achieved its objective* | The [intervention] has improved [behaviour/ condition/ clinical outcome]:   \| Strongly disagree \| Disagree \| No opinion \| Agree \| Strongly agree \| \| --- \| --- \| --- \| --- \| --- \| \| 1 \| **2** \| **3** \| **4** \| **5** \| | This item should relate to the primary outcome variable in the main trial/study, thus the item will be intervention specific. E.g. quality of life, walking ability |
| **Intervention coherence**  *The extent to which the participant understands how the intervention works* | It is clear to me how [intervention] will help [manage/ improve] my [behaviour/ condition/clinical outcome]   \| Strongly disagree \| Disagree \| No opinion \| Agree \| Strongly agree \| \| --- \| --- \| --- \| --- \| --- \| \| 1 \| **2** \| **3** \| **4** \| **5** \|   **Please tell us more about your views* | *In some studies, researchers may want to include an option for participants to provide more information for this item.    This will depend on the resources of the study for analysing qualitative data and whether having more information will be relevant. |
| **Self -efficacy**  *A participant’s confidence that they can perform behaviour(s) required to participate in the intervention* | How confident did you feel about [*behaviour i.e. engaging with*] [intervention]?   \| Very unconfident \| Unconfident \| No opinion \| Confident \| Very confident \| \| --- \| --- \| --- \| --- \| --- \| \| 1 \| **2** \| **3** \| **4** \| **5** \| |  |
| **Opportunity costs**  *The benefits, profits or values that would have to be given up to engage with the intervention* | [Behaviour i.e. *engaging in]* [intervention] interfered with my other priorities   \| Strongly disagree \| Disagree \| No opinion \| Agree \| Strongly agree \| \| --- \| --- \| --- \| --- \| --- \| \| 1 \| **2** \| **3** \| **4** \| **5** \| |  |
| **General acceptability** | How acceptable was the [intervention] to you?   \| Completely unacceptable \| Unacceptable \| No opinion \| Acceptable \| Completely acceptable \| \| --- \| --- \| --- \| --- \| --- \| \| 1 \| **2** \| **3** \| **4** \| **5** \| | The general acceptability item has been included as in some instances, an overall acceptability item may be useful and to allow for researchers to explore which of the 7 TFA constructs influences/ drives participants’ general acceptability judgment. |

**Analysis of TFA items:**

1. Items assessing burden and opportunity costs should be reverse scored so that a higher score always indicates higher acceptability
2. To generate a single acceptability score: (a) Compute the total mean score of the 7 TFA items, or (b) use the score for the General acceptability item. These two scores are likely to be positively, but not perfectly, correlated as score (a) assumes an equal weighting for each TFA construct. This may not always be the case. For example, for a life-threatening condition, responders may weight ‘perceived effectiveness’ more highly than ‘burden’ or ‘opportunity costs’.
3. Use a correlation matrix to compare the relationships between scores for each TFA item and the General acceptability item.
4. Further analyses will be dependent on the Research question under investigation, e.g.
   1. Comparing pre- and post-intervention acceptability: paired sample t-test
   2. Comparing the acceptability of two different interventions, delivered to two groups: independent samples t-test
   3. Comparing the prospective acceptability of one intervention after two groups of patients have been provided with different versions of a patient information leaflet: independent samples t-test.
